# Tubular Membrane Coupled with Marine Waste-Derived Hybrid Adsorbent for Textile Micropollutant Removal and Photochemical Regeneration

**DOI:** 10.3390/membranes16030110

**Published:** 2026-03-19

**Authors:** Rania Chihi, Mouna Ibn Mahresi, Fadhila Ayari, Lamjed Mansour, Amel Ben Othman

**Affiliations:** 1 Carthage University, Faculty of Sciences of Bizerte, LR 05/ES09 Laboratory of Applications of Chemistry to Resources and Natural Substances and to the Environment (LACReSNE), Zarzouna 7021, Tunisia; 2 Lorraine University, Reactions and Process Engineering Laboratory (UMR 7274), UMR CNRS 7274, BP 20451, 54001 Nancy, France; 3 Department of Zoology, College of Science, King Saud University, P.O. Box 2455, Riyadh 11451, Saudi Arabia; 4 Strasbourg University, School of Chemistry Polymers Materials, Molecular Design Laboratory, EPCM-ULP-CNRS, 25 Rue Becquerel, 67087 Strasbourg, France

**Keywords:** bentonite tubular membrane, treatment of textile wastewater, adsorption process, clam shell adsorbent, photochemical regeneration, circular economy, thermal regeneration, eco-friendly ceramic filters, hybrid filtration–adsorption

## Abstract

The development of sustainable ceramic membranes remains a major challenge for advanced wastewater treatment, particularly regarding the trade-off between mechanical durability and the removal of dissolved micropollutants. While bentonite membranes offer high stability, they often lack the selective adsorption sites required for complex effluents, and the recovery of high-capacity powder adsorbents remains technically prohibitive. This paper addresses these gaps by developing an integrated hybrid system that combines eco-friendly bentonite-based tubular membranes with regenerable clam shell-derived adsorbents. The membranes were synthesized using natural plasticizers and binders with optimization at a sintering temperature of 1000 °C yielding an average pore size of 1.7 µm, a high flexural strength of 24.06 MPa, and a permeability of 525 L h^−1^ m^−2^ bar^−1^. To enhance the performance, clam shell powder was integrated as a functional adsorbent layer. When applied to real textile effluent from a jeans washing plant, this integrated process achieved superior removal efficiencies: 85.6% COD, 86.5% BOD_5_, 86.5% TSS, and 96.5% color. A key scientific contribution of this paper is the successful application of a photochemical regeneration approach, which ensures complete adsorbent recovery and maintains membrane flux, directly supporting circular economy objectives. These results demonstrate that combining low-cost ceramic scaffolds with marine waste-derived materials provides a unique, efficient, and green solution for the scalable treatment of industrial wastewater.

## 1. Introduction

The volume of liquid waste discharged from municipal wastewater treatment plants (WWTPs) has increased significantly in recent decades. Negative environmental impacts are often associated with the textile dyeing industry, mainly due to the discharge of wastewater, which impairs the quality of the aquatic environment by changing its color and creating conditions for eutrophication, low reoxygenation, and decreased solar light penetration [1].

Moreover, the rapid evolution of society has led to an increased demand for higher water quality across all sectors: urban consumption, agriculture, industry, etc. Therefore, it has become essential to maximize wastewater treatment and minimize discharges from water treatment plants. Many traditional methods used for wastewater treatment are unable to completely remove toxins, pesticides [2], pharmaceutical residues [3], arsenic [4] and herbicides from contaminated water. The need to develop and implement more efficient and economically viable treatment technologies has become increasingly urgent, which is today dictated by resource scarcity, tougher environmental standards and the need to reduce energy costs.

Given the disadvantages of traditional wastewater treatment processes and the limitations of single-stage processes, interest in hybrid processes has increased substantially, which is driven by the need for overall process optimization and cost reduction [5,6]. Recent scientific studies have focused on combining low-pressure membrane processes, such as microfiltration (MF) and ultrafiltration (UF), with adsorption, coagulation, and photocatalysis [1,6,7,8,9]. At the heart of this technological transition, membrane filtration stands out as a reference solution to meet the most stringent water quality requirements. Membrane filtration, although effective in removing micropollutants, has long been hindered by its energy costs. Today, the research is oriented toward the development of eco-friendly materials with rigorous mechanical resistance. In addition to the utilization of bentonite clay and clam shell as feedstocks for membrane filtration and adsorption, which are known as inorganic materials that are environment friendly, renewable, and abundant, high mechanical strength (clays) and low-cost materials will add additional economic value to their place in the global marketplace. These materials can further maximize the lifespan of installations, ensure the economic profitability of separation process, and increase the potential and performance of the hybrid process treatment.

The purpose of this laboratory study was to use a combination of strategies to minimize the weaknesses in existing processes. Membrane processes, as well as adsorption and photocatalytic degradation, are alternatives to conventional treatment methods. The series configuration of adsorption and membrane filtration has been the most commonly used method for treating refinery wastewater, providing an effective way to improve the quality of effluent. However, the combination of membrane filtration and adsorption is rarely applied in effluent treatment. The main advantages of this hybrid process are the enhancement of membrane performance and the achievement of a highly efficient wastewater treatment technique. Three common processes were investigated to determine the optimum configuration for wastewater treatment at SITEX, which is a Tunisian textile factory. It is important to note that in this paper, we aimed to develop an innovative adsorbent for use in the adsorption process for treating effluent.

## 2. Materials and Methods

### 2.1. Raw Material

Bentonite clay was our starting material. It was collected from Gabes (southern Tunisia). The clay was crushed for 20 min with the assistance of a motor crusher (Retsch GmbH, Haan, Germany) and then sieved to a fine powder with a mesh size of 100 µm.

The sorbent used is a clam shell powder from the lake of Menzel Jemil, which is a city located about sixty kilometers north of Tunis in the governorate of Bizerte.

The clam was dried at room temperature for 4 days. The clam shell was ground using an alumina ball mill for six hours at a speed of 250 rpm. The white powder obtained was more or less heterogeneous in size. To better homogenize this granulometry, this powder was sieved and recovered at the fraction of 63 µm.

The plasticizer, methocel, was purchased from Dow Chemical Company (Zurich, Switzerland). The Amijel was used as dispersant in the paste and purchased from Roquette Italia S. p. A (Cassano Spinola, Italy). The binder used was starch, which was purchased from RG 03408 Cerestar (Paris, France). The solvent used was distilled water.

### 2.2. Membrane Preparation

The preparation of a ceramic membrane from natural material with a tubular configuration has been carried out.

To obtain a porous and resistant membrane with clayey features, the addition of organic additives is necessary. Several tests were conducted to prepare pastes using different types and amounts of additives, including Amijel as a plasticizer in varying doses (4–10 wt%), Methocel as a binder in different quantities (4–10 wt%), and starch as a porosity agent in varying concentrations (4–10 wt%) with varying proportions of solvent.

The optimal formulation for the paste used in the development of tubular ceramic membranes was determined and thoroughly detailed (Table 1).

Once the paste was prepared, it was shaped into a cylindrical block. After aging for two days, the paste was extruded at a screw speed of 0.02 m·min^−1^ to obtain tubular membranes with an external diameter of 5 mm (±0.01), an internal diameter of 1.5 cm (±0.01), and a length of 4.5 cm (±0.01).

**Figure 1:**
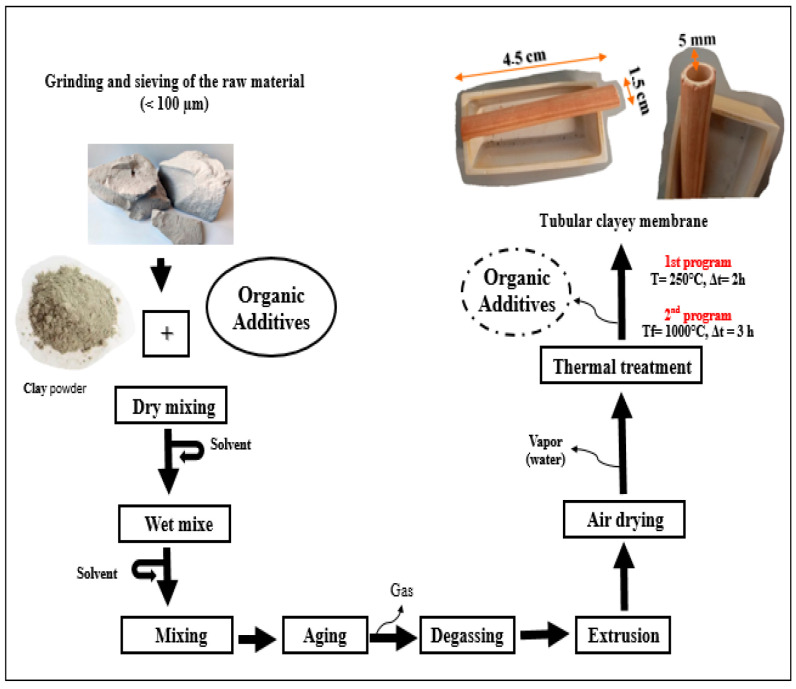
Process used to synthesize clayey membrane.

At the die outlet, the extruded tubular membranes were placed in a humidity-controlled environment during the drying stage to prevent cracking and ensure structural stability. Finally, the dried ceramic membranes were sintered at 1000 °C to improve their porosity, structural integrity, and mechanical properties. The synthesis procedure of the clayey membranes is described in detail in Figure 1.

### 2.3. Study Area

**Figure 2:**
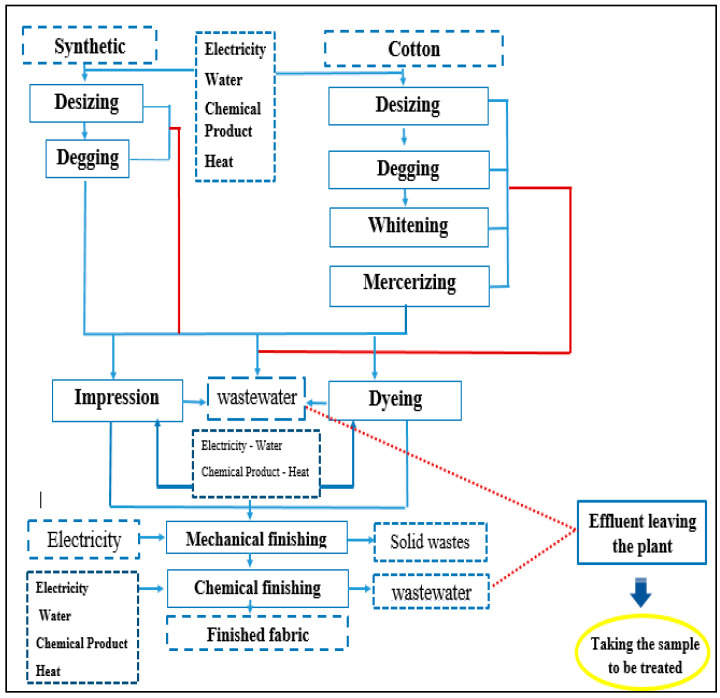
Activity of the company SITEX.

This paper was conducted by relying on effluent from a textile factory (SITEX) located in Ksar Hilal–Tunisian Sahel–Tunisia. This company specializes in the preparation process, indigo dyeing and finishing (Figure 2). These activities consume large quantities of water (average of 600–800 m^3^ d^−1^) and consequently generate polluted effluents that are collected in a single homogenization basin before final treatment by biological process.

The raw textile effluent was collected from the homogenizer at the exit of the plant, after which it was loaded with dye, sodium chloride, sodium dithionite (Na_2_S_2_O_4_), enzymes, surfactants, soda and emulsifying agents.

The sample is stored in sealed containers and kept refrigerated until the time of discoloration treatment.

The standards for wastewater discharge are becoming increasingly strict, setting physicochemical and biological quality indicators. Investigations are conducted to determine the evolution of the effluent’s characteristics before treatment.

The main physicochemical and biological characteristics of the effluent sample used in this study are presented in detail (Table 2). The effluent studied is dark blue, indicating the presence of a significant load of dyes such as indigo, sulfur dyes, vat dyes, and other chemicals like surfactants, wetting agents, and dispersants. The effluent, with an alkaline pH of 12.4, must be neutralized to comply with Tunisian discharge standards (NT 106.02 (1989) [10].

A conductivity of about 12,372 µS·cm^−1^ at 22 °C explains the high presence of ions. High values of chemical oxygen demand (COD) (1400 mg·L^−1^) and biological oxygen demand (BOD5) (400 mg·L^−1^) indicate the high level of organic and mineral matter load. The maximum adsorption peak of this effluent is around 594 nm. The values obtained are very high compared to the standards authorized by the legislation (Table 2).

### 2.4. Membrane Characterization

Different techniques were used to characterize raw materials and synthesized membranes. The X-ray fluorescence was used to determine the chemical composition or elements present in the sample used. XRF measurements were performed using a commercial instrument (ARL 9900, Thermo Fisher Scientific, Waltham, MA, USA) with a monochromatic radiation K_α_ of cobalt (λ = 1.788996 Å). The phases present in the powder composition were analyzed by an X-ray diffractometer using a Panalytical X’Pert High-score plus diffractometer with Cu Kα radiation (λ = 1.5406 Å, Malvern Panalytical, Almelo, Netherlands). Particle size distribution was measured using a Microtrac SYNC laser diffraction system (Retsch GmbH, Haan, Germany; measuring range: 0.01 µm–4 mm) correlated with a computer. Thermogravimetric analysis (TGA) was conducted using a Mettler Toledo TGA/SDTA 851 apparatus (Mettler Toledo, Greifensee, Switzerland). Approximately 15 mg of samples was heated from ambient to 900 °C at a constant rate of 5 °C/min under nitrogen. Scanning electron microscopy (SEM) was performed using a Hitachi S-4800 microscope (Hitachi, Tokyo, Japan) to determine the surface morphology of the samples before and after treatment.

### 2.5. Filtration Process

#### 2.5.1. Methodology

The tubular membrane was tested on a laboratory pilot, using textile effluent from the dyeing industry. The pilot used was built in the laboratory entirely in stainless steel to avoid the risks of corrosion. It operates in batch mode with the circulation of the retentate ensured by a pump.

**Figure 3:**
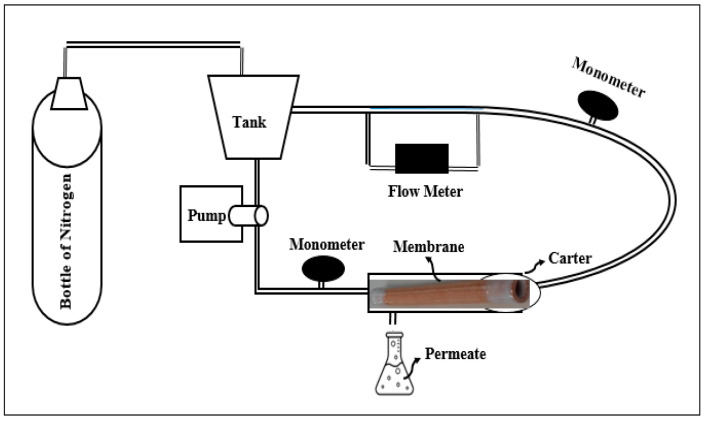
Schematic process used for the filtration process.

A housing is designed to accommodate tubular membranes with a length of 15 cm; however, several other lengths are available. The transparent casing allows for the visual monitoring of phenomena on the external surface of the membrane during operation. The working pressure inside the pilot is maintained by a compressed nitrogen cylinder, which is controlled by two sensors placed upstream and downstream of the membrane and regulated by a valve (Figure 3).

#### 2.5.2. Experimental Protocol

The experimental protocol followed in the treatment consists of the following:

- Physicochemical analysis of the raw effluent;
- Membrane filtration;
- Physicochemical analysis of the filtrate followed by a comparative study with the real effluent.

After filtration, a cleaning cycle is necessary in order to regenerate the membrane used. Generally, the regeneration of commercial mineral membranes occurs according to a protocol using an alternation of basic and acid baths: the regeneration of the membrane was performed by back washing with 0.01 M NaOH solution followed by 0.01 M H_2_SO_4_ at 80 °C over three hours.

### 2.6. Adsorption Experiments

Adsorption experiments were carried out at room temperature (25 ± 2 °C). Approximately 9 g of clam shell was weighted into containers and brought into contact with 50 mL of permeate solution. The solutions were maintained under constant mechanical stirring during the necessary time to reach adsorption equilibrium (90 min). The resulting suspension were sampled and centrifuged for further chemical analysis of the supernatant.

### 2.7. Regeneration Experimental Protocol

The experiment is based on an adsorption/regeneration cycle. The adsorption of the textile effluent is performed to achieve the fixation of the effluent on the clam shell; then, the adsorbent is recovered to be put in advanced oxidation process conditions (regeneration).

#### 2.7.1. Operating Mode

The photochemical reactor was composed of 1 g of adsorbent saturated with adsorbate (clam shell + effluent) with 150 mL of distilled water. Then, the necessary quantities of Fe_2_SO_4_ catalyst and H_2_O_2_ oxidant are added, and the UV lamp is switched on.

The photo-regeneration is started and lasts 120 min. In the absence of UV irradiation, for the Fenton process, the duration is 4 h. At the end of the treatment, the samples are centrifuged and filtered on Millipore 0.45 μm filters for analysis. The concentration of the effluent existing in the reaction medium after Fenton and photo-Fenton regeneration is quantified by chemical oxygen demand (COD) measurements.

The experimental conditions for the regeneration phase are summarized as follows:

V(H_2_O_2_) = 1 mL, [Fe_2_SO_4_] = 1 mmol L^−1^, V = 150 mL, pH = 7, regeneration time t = 2 h in the presence of UV irradiation and 4 h in the absence of UV and T = 25 °C.

#### 2.7.2. Irradiation Device

**Figure 4:**
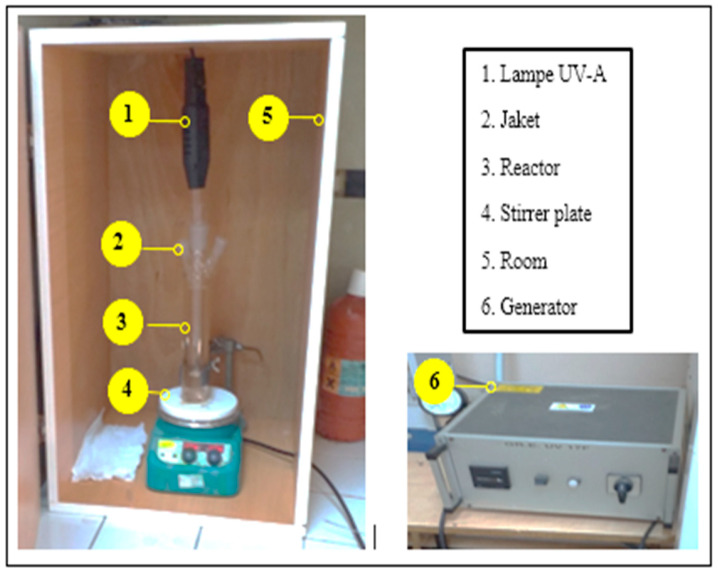
Irradiation device.

Adsorbent regeneration tests using the photo-Fenton process were performed in a 150 mL photoreactor equipped with a low-pressure mercury vapor UV-A lamp (λ = 350 nm) operating near room temperature (Figure 4). The UV lamp is shielded from the solution by a quartz tube placed axially. The reactor is placed in a chamber ensuring total darkness to protect itself from the emitted UV radiation on the one hand and to avoid any reaction involving light on the other hand.

## 3. Results and Discussion

### 3.1. Characterization of Raw Material

#### 3.1.1. XRD Analysis

**Figure 5:**
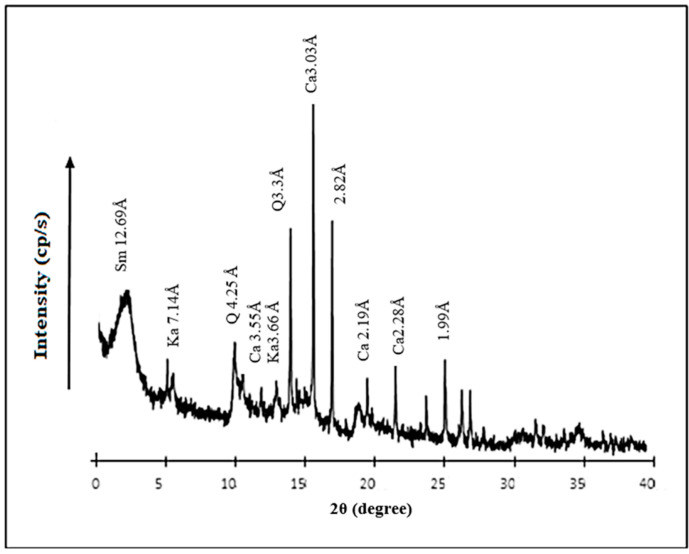
XRD pattern of clay sample (Sm = smectite, Ka = kaolinite, Q = quartz, Ca = calcite).

X-ray diffraction of clay suggest that it is a smectite sodic sample (2θ = 12.69 Å) associated with quartz (2θ = 20.89° and 26.65°), calcite phases (2θ = 23.10°, 26.65°, 36.02° and 39.45°) and kaolinite fraction (2θ = 12.38°, 24.96°) (Figure 5) [11].

#### 3.1.2. XRF Analyses

The data present high silica content (SiO_2_) of about 70.5 wt%, which consisted of clays and quartz. The alumina content (Al_2_O_3_) is 14.31 wt% corresponds to aluminous clays. The calcium carbonate content in the samples was 2.4 wt%. Some elements were present in trace amounts in the raw material, such as iron oxide, magnesium and sodium (Table 3).

#### 3.1.3. TGA Analysis

**Figure 6:**
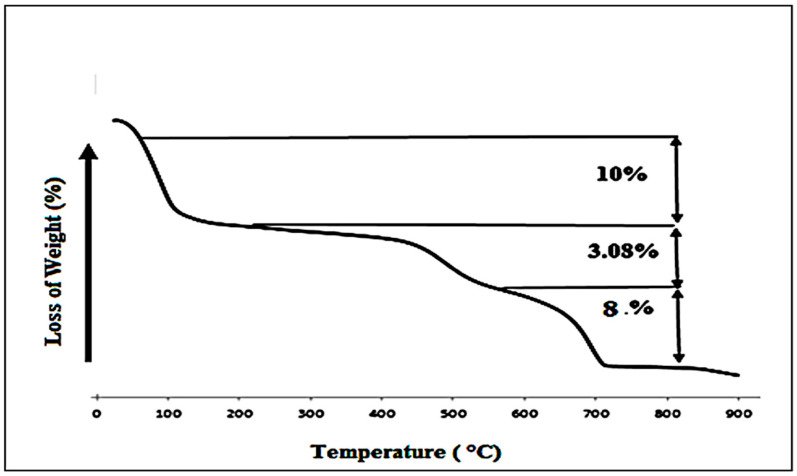
TGA thermogram of clay.

The clay TGA curves present three mass losses: the first mass loss (10%), from 323 K to 473 K, was attributed to moisture and water interlayer (Figure 6). The second mass loss (3.08%) occurs from the 473 K to 873 K temperature range related to structural water loss. The final loss mass (8%) was attributed to the decarbonization of clay.

#### 3.1.4. Particle Size Distribution Analyses

**Figure 7:**
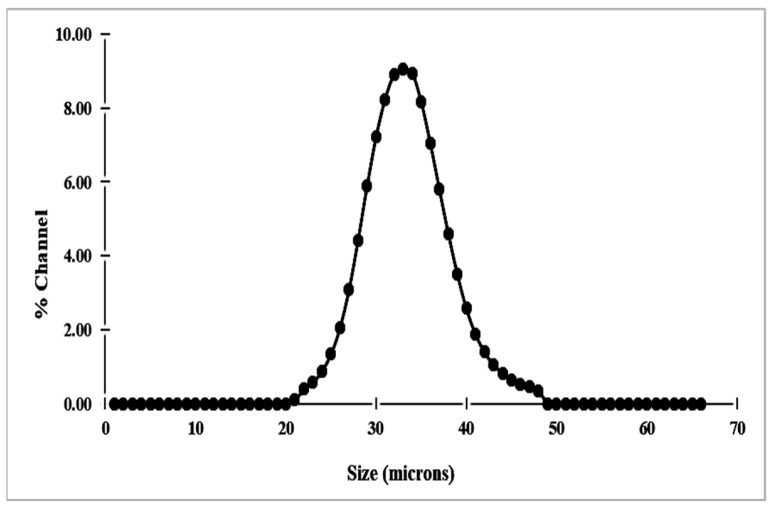
Particle size distribution of Bentonite.

The distribution of existing particles shows an asymmetrical shape with particle sizes ranging between 30 and 38 µm (Figure 7). Based on these results, it can be inferred that the raw material has a good fineness, which could affect the compact density and porosity of the elaborated membranes.

### 3.2. Characterization of Ceramic Membrane

**Figure 8:**
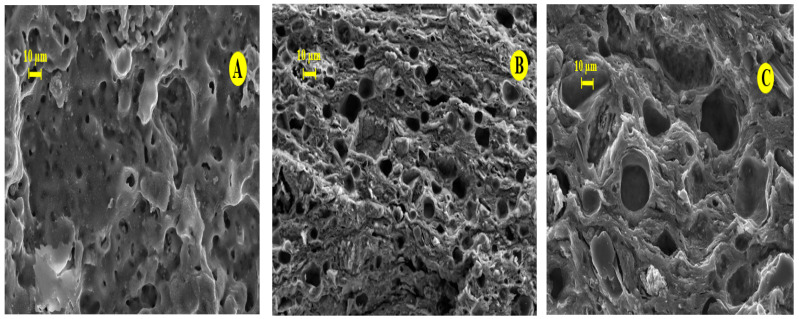
SEM micrographs of membrane, screened at 60 μm, at different temperatures: (**A**) membrane sintered at 1100 °C, (**B**) membrane sintered at 1000 °C, (**C**) membrane sintered at 950 °C.

**Figure 9:**
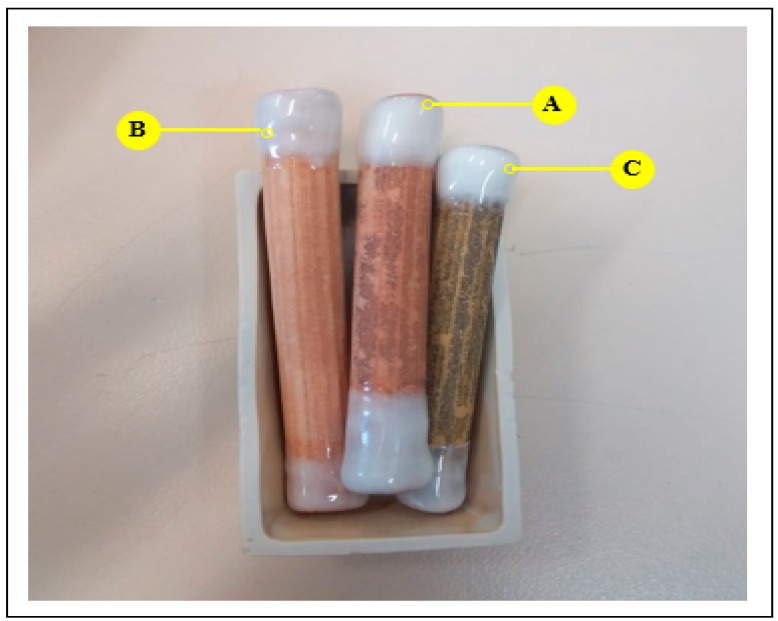
Three ceramic membranes sintered at different temperatures: (A) membrane sintered at 1100 °C, (B) membrane sintered at 1000 °C and (C) membrane sintered at 950 °C.

According to the results obtained in our previous work [12], the development of a tubular ceramic membrane was carried out using an extrusion process followed by sintering at different temperatures (800, 950, 1000 and 1100 °C) (Figure 8 and Figure 9).

Grain consolidation begins between 800 °C and 950 °C; however, the mechanical strength remains limited at these temperatures. At 1000 °C, a marked improvement in microstructural organization is observed. The grains become closely packed and intergranular contacts are well established, ensuring sufficient cohesion and structural stability. This stage corresponds to the onset of effective sintering, where grain growth is controlled and interparticle bonding becomes dominant.

At 1100 °C, densification intensifies due to grain welding and coarsening phenomena. Smaller grains progressively disappear in favor of larger ones, leading to porosity reduction and the formation of a nearly monolithic structure. In order to preserve adequate porosity while ensuring sufficient mechanical resistance, 1000 °C was selected as the optimal sintering temperature. SEM observations confirm a homogeneous and uniform surface morphology without significant agglomeration (Figure 8).

Under these conditions, the membrane exhibits an average pore diameter of 1.7 µm, a flexural strength of 24.06 MPa, and a water permeability of approximately 525 L·h^−1^·m^−2^·bar^−1^. Chemical stability tests performed using 0.2 M HCl followed by 0.5 M NaOH for 96 h demonstrate excellent resistance with no visible degradation or structural alteration.

From a functional perspective within the hybrid treatment system, the relatively large pore diameter (1.7 µm) indicates that the membrane primarily operates as a clarification step. While individual dissolved dye molecules are significantly smaller than the membrane pores and can pass through, then suspended solids, colloidal matter, and dye aggregates are effectively retained. This pre-filtration reduces the turbidity and particulate load before the adsorption stage.

The subsequent adsorption step using clam shell material is therefore responsible for removing the dissolved fraction that cannot be intercepted by microfiltration. In this configuration, the membrane and the adsorbent perform complementary roles: the membrane ensures the physical separation of particulate species, whereas adsorption targets molecular-scale pollutants through surface interaction mechanisms.

Such a distribution of functions explains the improved overall efficiency of the hybrid process and supports the existence of a synergistic effect rather than a simple sequential operation. The membrane reduces the burden on the adsorbent surface, which may contribute to improved operational stability and reduced fouling during treatment.

SEM images confirm that there is no agglomeration, and the surface remains uniform and homogeneous, indicating that the chosen temperature meets the material’s desired specifications (Figure 8).

**Figure 10:**
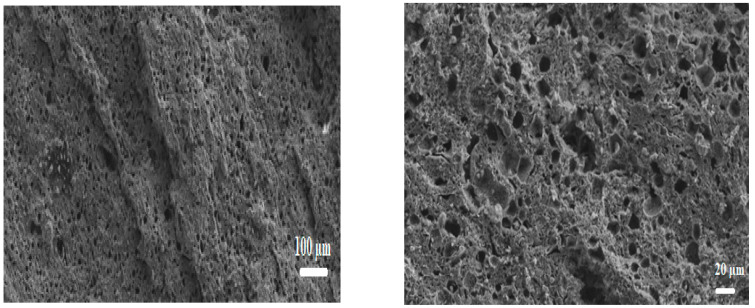
SEM micrographs of membrane sintered at 1000 °C.

SEM images show that the grains become sufficiently close, and the internal reorganization of each is well established, forming a strong roughness (Figure 10). At this stage, the presence of intergranular contacts can be detected, which are sufficient to ensure the cohesion of the ceramic. The beginning of the sintering process marks the end of the grain coarsening and the formation of the joints.

For regeneration, the membrane was treated with 0.2 M HCl followed by 0.5 M NaOH solution at ambient temperature for more than 96 h. The regenerated membrane showed good stability in both acidic and alkaline environments with no noticeable degradation, aging, or color change. These properties confirm that the membrane provides both structural stability and sufficient permeability for its role as a pre-filtration step in the hybrid system, ensuring particulate removal while maintaining operational robustness prior to the adsorption stage.

### 3.3. Characterization of the Adsorbent

#### 3.3.1. Analysis by Infrared Spectrometry

**Figure 11:**
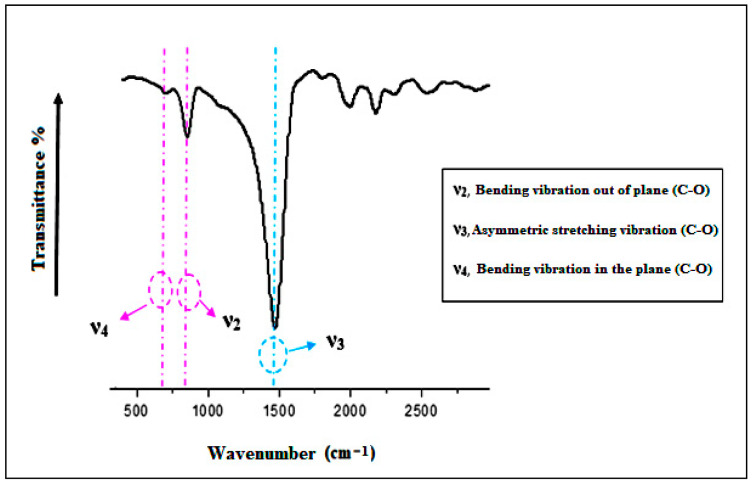
Infrared spectrum of clam shells.

The infrared spectrometry analysis of the adsorbent indicates that the characteristic bands located at 1420, 874, and 713 cm^−1^ are attributed to the antisymmetric stretching vibrations (ν_3_), out-of-plane bending vibrations (ν_2_), and in-plane bending vibrations (ν_4_) of the carbonate group (C–O), respectively [13,14] (Figure 11).

#### 3.3.2. X-Ray Diffraction

**Figure 12:**
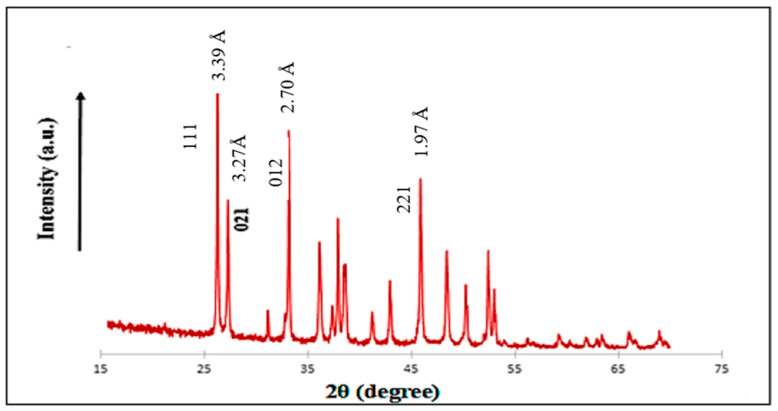
X-ray diffractograms of clam shell.

The result obtained in the diffractograms gives information on the structure and mineralogical composition of the clam shell (Figure 12). The lines present at (111), (021), (012), and (221) correspond to 3.39 Å (2θ = 26.23°), 3.27 Å (2θ = 27.26°), 2.70 Å (2θ = 33.13°), and 1.97 Å (2θ = 45.86°), respectively, and they are indicative of the existence of calcite [15].

#### 3.3.3. Thermogravimetric Analysis (TGA)

**Figure 13:**
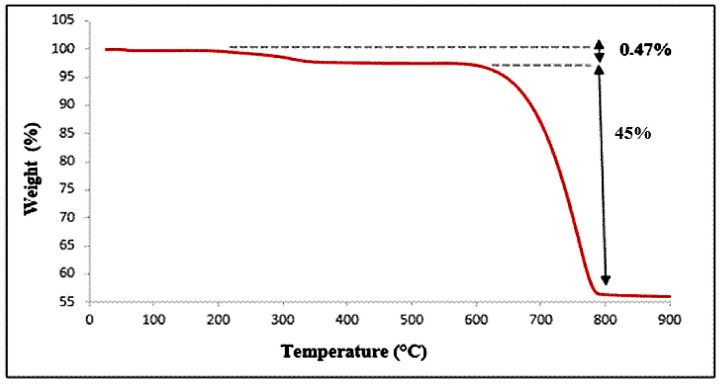
Thermogravimetric analysis curve of clam shells.

The thermal behavior of the clam shell was investigated by thermogravimetric analysis (TGA) using a temperature program ranging from 25 °C to 900 °C at a heating rate of 5 °C·min^−1^ (Figure 13). This analysis was conducted to monitor the thermal decomposition process of the material. The mass loss recorded during heating resulted in a thermogram showing two main weight-loss stages (Figure 13).

The first mass loss (approximately 0.47%) was observed between 320 and 610 °C and is mainly attributed to the degradation of residual organic matter [16].

**Figure 14:**
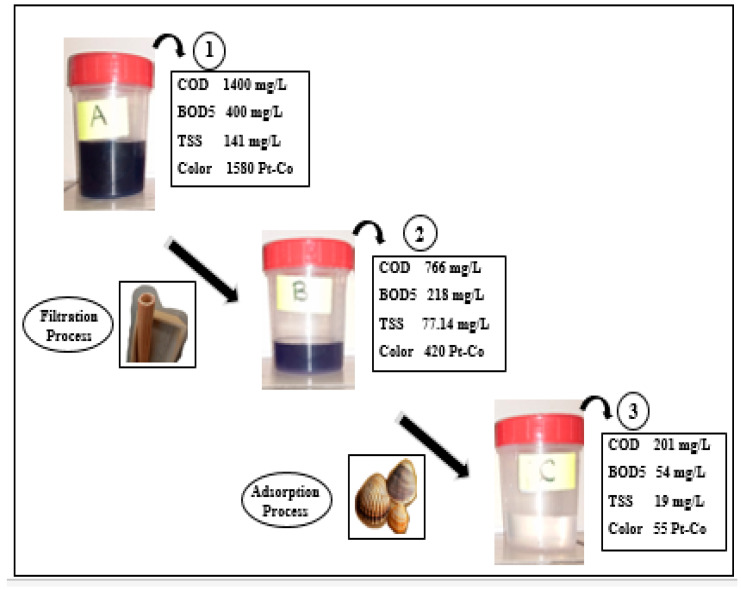
Schematic diagram of the hybrid treatment system.

A second significant mass loss (around 45%) occurred between 700 and 800 °C (Figure 14), corresponding essentially to the thermal decomposition of calcite (CaCO_3_). This mineralogical transformation leads to the formation of solid calcium oxide (CaO) and the release of carbon dioxide (CO_2_) gas. The reaction mechanism is presented as follows [17,18]:

(1) $${\mathrm{CaCO}}_{3 (s)}\;\overset{∆T}{\to }{\mathrm{CaO}}_{\mathrm{(s)}}+{\mathrm{CO}}_{2 (g)}$$

## 4. Application

### 4.1. Filtration and Adsorption Studies

The optimum operating condition of the ceramic membrane was obtaining from our previous paper [16]. The performance of the integrated filtration followed by the adsorption treatment process was investigated under different loads of COD, BOD_5_, TSS and color.

The removal rates and concentrations of COD, BOD_5_, TSS and color before and after subjecting to consecutive treatment operations, including the filtration and adsorption process, are detailed in Figure 14 and Table 4. The use of the filtration process followed by adsorption to the treatment system is foundational to the system’s performance in removing COD, BOD_5_, TSS and color.

**Figure 15:**
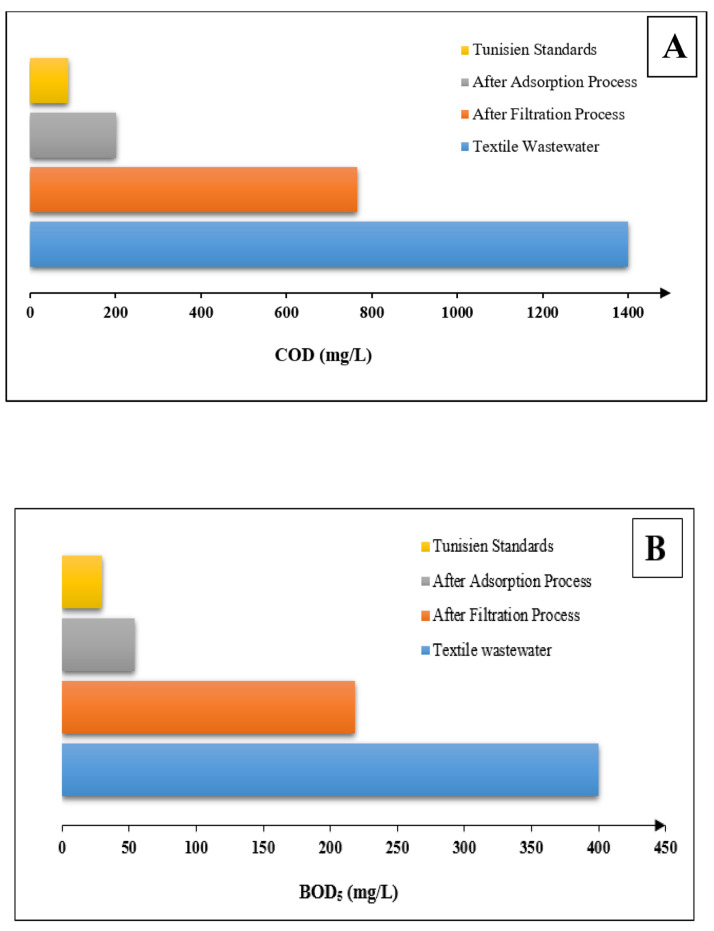

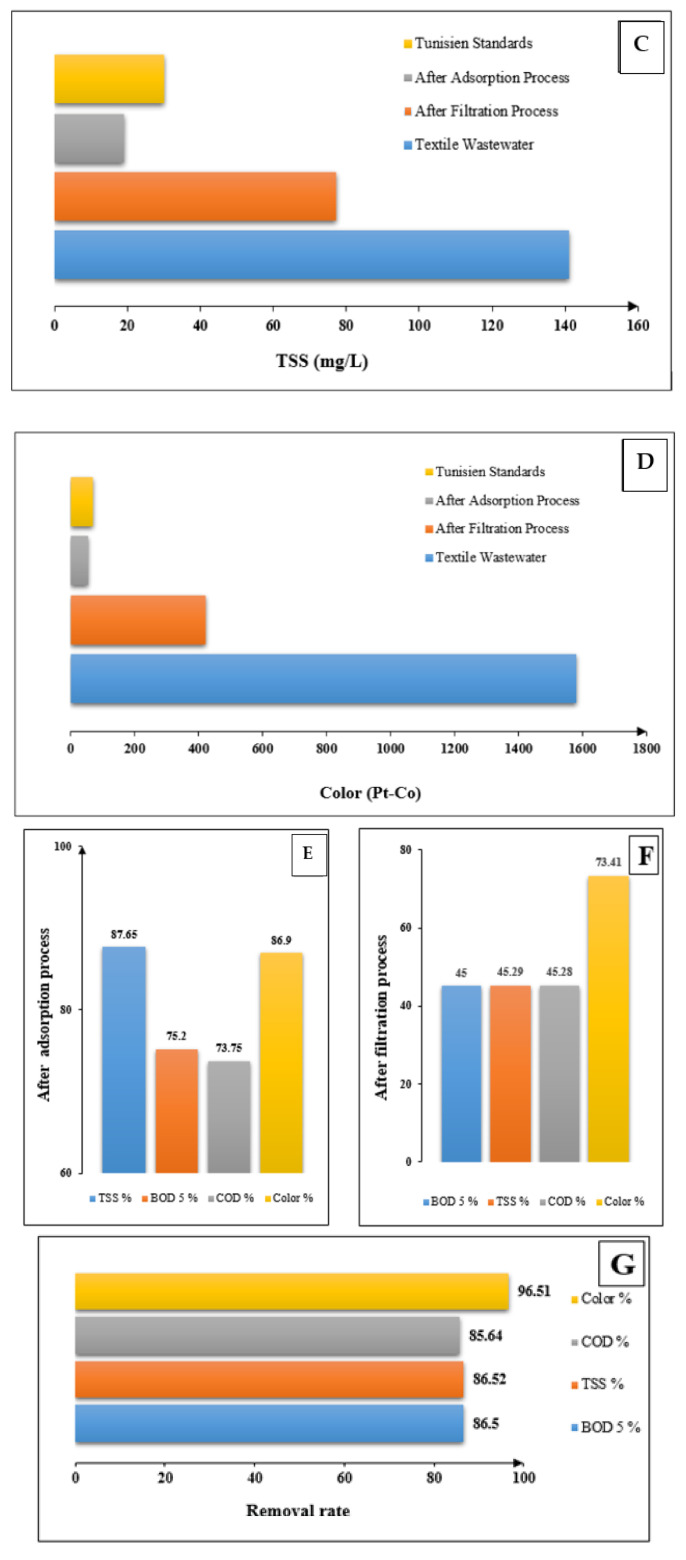
Experimental results after filtration followed by an adsorption treatment process with different loads of the following: (**A**) COD, (**B**) BOD5, (**C**) TSS, (**D**) color, (**E**) removal rates after adsorption treatment process, (**F**) removal rates after filtration treatment process and (**G**) total removal rates.

According to the results obtained in Figure 15A, the COD removal efficiency improved from 45.28% to 73.75% after applying filtration and adsorption, respectively. For TSS, the removal efficiency increased from 77.14% to 87.65% following these processes (Figure 15C). In accordance with the results achieved in Figure 15E, the color concentrations presents a significant decrease with an increase in removal efficiency from 73.41% to 86.9%. The removal rates for BOD_5_ were increased from 45% to 75.2% (Figure 15B) after filtration and adsorption, respectively.

The results obtained showed that the average removal reached 86.5% for BOD_5_, 86.52% for TSS, 85.64% for COD and 96.51% for color, which is a result of the efficiency of the used materials and their ability to break down organic constituents (Figure 15).

### 4.2. Surface Charge Characteristics and Adsorption Mechanism

Clam shell-derived materials are widely reported in the literature as CaCO_3_-rich biosorbents predominantly composed of calcite. Their surface chemistry is characterized by exposed Ca^2+^ sites and carbonate groups (CO_3_^2−^), which can participate in adsorption through electrostatic interactions and surface complexation mechanisms.

In this paper, the point of zero charge (PZC) of the clam shell powder was experimentally determined and found to be pH 8.8. The measurement was carried out using a zeta potential analyzer (electrophoretic light scattering technique, temperature 25 °C, KCl 10^−3^ M as background electrolyte). This value is consistent with those commonly reported for calcite-based materials derived from marine shells.

Since the measured effluent pH is 12.4, which is significantly higher than the PZC, the adsorbent surface is expected to be predominantly negatively charged due to the deprotonation of surface hydroxyl groups (≡Ca–OH → ≡Ca–O^−^). Under these strongly alkaline conditions, electrostatic attraction between negatively charged surface sites and anionic species is not favored.

The investigated wastewater originates from an indigo dyeing process. Indigo (Indigo) is applied under strongly alkaline conditions in its reduced (leuco) form. Therefore, the adsorption mechanism cannot be attributed solely to electrostatic interactions.

The removal process is more plausibly governed by a combination of the following:

(i) Surface complexation, involving the coordination between surface Ca^2+^ sites and electron-donating groups of dye molecules or other organic constituents;
(ii) Calcium-mediated interactions and possible co-precipitation, resulting from the partial dissolution of CaCO_3_ under alkaline conditions;
(iii) Physical adsorption and pore diffusion, facilitated by the biogenic porous structure of the shell material;
(iv) Hydrophobic interactions due to the aromatic structure of indigo molecules.

Considering the complex composition of real textile effluents, the overall removal efficiency is likely controlled by the synergistic contribution of these physicochemical mechanisms rather than by purely electrostatic attraction.

### 4.3. Regeneration

The regeneration of the natural adsorbent was carried out by a photochemical method using two catalytic oxidation processes: the Fenton process and the photocatalytic oxidation process (photo-Fenton).

**Figure 16:**
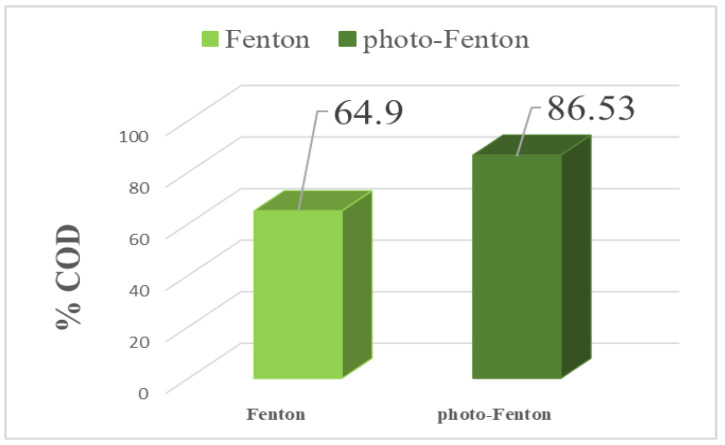
Effect of Fenton and photo-Fenton as different regeneration modes on the degradation of the effluent stored in the clam shell.

The performance of the regenerated adsorbent is presented in Figure 16. The results indicate that in the absence of UV irradiation and in the presence of the oxidant H_2_O_2_, the degradation efficiency reached 64.9% after 4 h of reaction. This behavior can be explained by the reaction between hydrogen peroxide (H_2_O_2_) and Fe^2+^ ions present in the reaction medium, leading to the generation of hydroxyl radicals (•OH). The degradation of the desorbed effluent is therefore mainly attributed to the strong oxidative action of these •OH radicals (Equation (2)).

**Figure 17:**
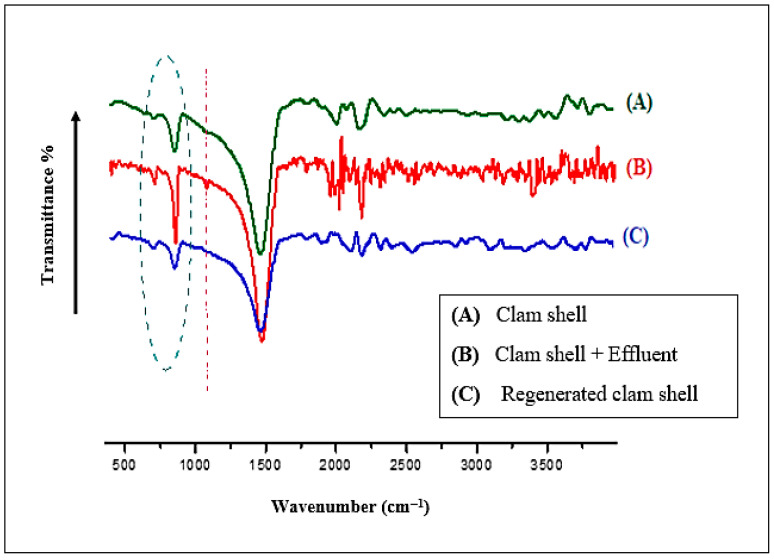

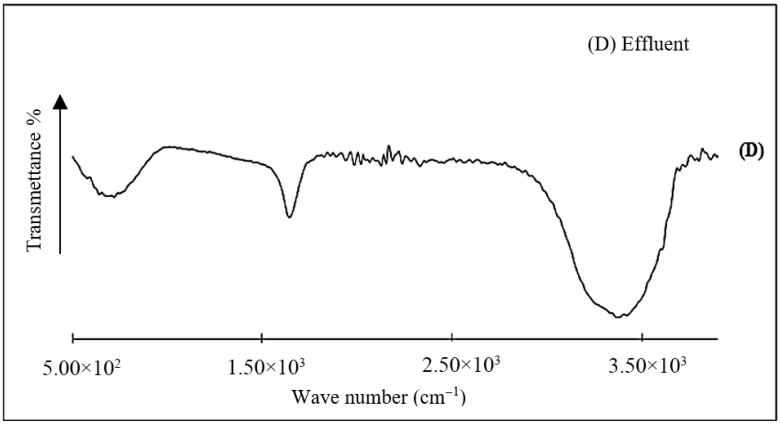
Infrared spectra of (A) clam shell powder, (B) clam shell powder + effluent, (C) regenerated clam shell powder, and the studied effluent (D).

When the solution is subjected to UV irradiation in the presence of H_2_O_2_, the rate of COD degradation reaches 86.53% (Figure 17). This phenomenon can be attributed to the synergy between UV irradiation and Fenton reagents in the reaction medium, where Fe^2+^ and H_2_O_2_ are present, which is a process commonly referred to as the photo-Fenton process (Equations (3) and (4)).

The results obtained highlight the essential role of UV irradiation in the regeneration of the adsorbent studied, demonstrating the effectiveness of this process.

(2) $${\mathrm{Fe}}^{2+}+H_{2}O_{2}\;\to {\mathrm{Fe}}^{3+}+{\mathrm{HO}}^{•}+HO^{-}$$

(3) $${\mathrm{Fe}}^{2+}H_{2}O_{2}+h\nu \;\to {\mathrm{Fe}}^{3+}+{\mathrm{HO}}^{•}+HO^{-}$$

(4) $$H_{2}O_{2}+h\nu \;\to {\mathrm{2 HO}}^{•}$$

#### Infrared Spectrometry Analysis

The infrared spectroscopy data presented illustrate the regeneration process of clam shell powder before and after effluent adsorption (Figure 17). The IR spectra of the adsorbent (clam shell powder) reveal the presence of specific bands corresponding to the functional groups of the effluent. After completing the treatment cycle, these bands disappear, indicating successful adsorbent regeneration. The resulting IR spectrum (Graph C, Figure 17) is identical to that of the original material.

### 4.4. Comparison with Literature

As shown in Table 5, our proposed treatment process, which includes three stages (filtration, adsorption, and photo-Fenton) demonstrates significantly higher removal rates compared to the other single-stage and combined systems listed. Additionally, a key challenge in the widespread implementation of these single-stage and combined treatment systems is the elevated costs associated with chemicals and the overall energy consumption. Therefore, based on the results obtained, it can be concluded that the hybrid treatment processes represent an effective technology for removing stable, persistent organic compounds such as COD, TOC, and BOD_5_ to levels below the permissible discharge limits.

## 5. Combined Systems Economic Evaluation

Along with efficiency evaluation, cost estimation is an important parameter. The overall costs of the treatment process include the direct cost items such as raw material consumption, reagents, labors charge, energy consumption cost and mechanical and electrical instruments, whereas indirect costs include maintenance and other costs. Table 6 summarizes the used calculation results. The direct costs were estimated to be 74.39 USD/m^3^ taking into consideration the cost estimated for the labor charge, 37.5 USD/m^3^. The mechanical and electrical instruments were estimated as 24 USD/m^3^/year. The maintenance and others cost were considered to be 12 and 7 USD/m^3^/year.

## 6. Conclusions

In this work, three treatment processes—membrane filtration, adsorption, and photocatalytic oxidation—were evaluated for the treatment of textile wastewater from the SITEX factory in Tunisia. The tubular clay membranes prepared in this study showed appropriate structural and mechanical properties. The membrane sintered at 1000 °C, which was characterized by an average pore diameter of 1.7 µm, a flexural strength of 24.06 MPa, a water permeability of about 525 L·h^−1^·m^−2^·bar^−1^, and good chemical resistance, demonstrating stable performance during filtration experiments.

The adsorption process was carried out using clam shell as a natural and low-cost adsorbent rich in calcium carbonate. The combined treatment system achieved high removal efficiencies at pilot scale with average reductions of 86.5% for BOD_5_, 86.52% for TSS, 85.64% for COD, and 96.51% for color. These values clearly demonstrate the effectiveness of coupling membrane filtration and adsorption for textile wastewater treatment. The quality of the treated effluent complied with regulatory standards, supporting its potential reuse in irrigation of salt-tolerant crops or safe discharge into surface waters.

Regeneration of the adsorbent was investigated using Fenton and photo-Fenton processes. In the presence of H_2_O_2_ without UV irradiation, COD degradation reached 64.9% after 4 h of reaction. When UV irradiation was applied (photo-Fenton), the degradation efficiency increased to 86.53%, confirming the positive effect of photochemical activation. Infrared analysis further confirmed the restoration of the adsorbent after treatment.

Overall, the experimental results consistently support the proposed treatment strategy. The removal efficiencies obtained, together with the successful regeneration of the adsorbent, demonstrate the technical feasibility and sustainability of the integrated process for textile wastewater treatment at larger scale.

## Figures and Tables

**Table 1:** Chemical composition of the optimized formulation used for the clayey paste.

	wt, %	Raw Material	Methocel	Amigel	Starch
Sample					
Clayey membrane		84	4	4	8

**Table 2:** Average concentrations of the raw dyed textile effluent in the factory with the Tunisian standards.

Parameters	pH	Conductivity	COD	BOD_5_	TSS	Color	λ_max_	Chloride
**Units**	_	**µScm^−1^**	**mgL^−1^**	**mgL^−1^**	**mgL^−1^**	**pt-co**	**nm**	**mgL^−1^**
**Effluent**	12.4	12,372	1400	400	141	1580	594	2000
**Tunisian Standards** **(Public Hydraulic Domain)**	6.5–8.5	1000	90	30	30	70	_	600

**Table 3:** Chemical analysis of clay as starting material (fractions < 2 µm).

Weight wt%	Fe_(T)_	SiO_2_	CaO	Al_2_O_3_	Na_2_O	MnO	MgO	TiO_2_	P_2_O_5_	SO_3_	K_2_O	LOI
**clay**	5.15	70.5	2.4	14.31	-	-	2.89	0.2	-	0.21	0.52	9.01

**Table 4:** Summaries of concentrations of COD, BOD_5_, TSS and color before and after subjecting to consecutive treatment operations. Comparison for Tunisian standard.

	COD (mg/L)	BOD_5_ (mg/L)	TSS (mg/L)	pH	Color (Pt-Co)
**Raw Effluent**	**1400**	**400**	**141**	**11.5**	**1580**
**After Filtration Process**	**766**	**218**	**77.14**	**9**	**420**
**After Adsorption Process**	**201**	**54**	**19**	**7.3**	**55**
**Tunisian standard for disposal of industrial wastewater into river (Tunisian standard NT 106.002, 1989 [17])**	**90**	**30**	**30**	**6.5–8.5**	**100**

**Table 5:** Summaries of various processes used for the treatment of real effluents.

Process Used	Remarks	Wastewater/Dye	Color %	COD%	BOD_5_%	TSS%	References
**Photocatalytic degradation process**	TiO_2_ nanoparticles Sol–gel method	Real effluent	-	79	80	11	[19]
**Adsorption process**	Clam shell as adsorbent	Real textile	86.9	73.75	75.2	75.3	This Work
**Adsorption process**	Activated carbon as adsorbent	Real effluent	-	65	58	10	[19]
**Filtration process**	Clayey tubular membrane Extrusion	Real textile	73.41	45.28	45.2	45.2	This Work
**Filtration process**	PAN/PVP membrane PAN flat sheet nanofiltration Polyvinylpyrrolidone (PVP) as additive Dimethylformamide (DMF) as solvent Phase inversion method	Real effluent	-	86	83	92	[19]
**Fenton reaction**	Used in a sequential bath reactor	Synthetic cotton textile wastewater	99	88	83	-	[20]
		Synthetic polyester textile wastewater	99	91	91	-	[20]
**1. Coagulation/flocculation** **2. Adsorption** **3. Filtration**	1. Using FeCl_3_ 2. Using nZVI 3. Using micro zeolite		98.4	97.5	_	98	[21]
**1. Filtration** **2. Adsorption** **3. Photo-Fenton**	1. Using clayey membrane 2. Clam shell 3. Fe_2_SO_4_	Real textile	99	86.53	94.5	92.19	This work
**1. Membrane filtration** **2. Adsorption** **3. Photocatalyst**	1. Using PAN/PVP membrane 2. Using activated carbon as adsorbent 3. Using TiO_2_ nanoparticles	Real effluent	-	88	86	98	[19]
**1. Adsorption** **2. Membrane filtration** **3. Photocatalyst**	1. Using activated carbon as adsorbent 2. Using PAN/PVP membrane 3. Using TiO_2_ nanoparticles	Real effluent	-	89	87	96	[19]
**1. Adsorption** **2. Membrane filtration**	1. Powdered activated carbon (commercial CWZ30) as adsorbent 2. Polyacrylonitrile as UF membrane	organic carbon	92%	65%	-	-	[22]
**EC: Electrocoagulation** **EO: Electrooxidation**	EC + EO	Textile wastewater	97.5%	93.5%		97%	[23]
**Photo-Fenton process**	Using Fe_2_SO_4_	Real textile	99	86.53	59.25	42.10	This Work
**EC: Electrocoagulation** **EO: Electrooxidation**	EC + EO	Textile wastewater	-	90	87.0	-	[24]

**Table 6:** Economic evaluation of the combined treatment process.

**Quantity estimated**	200 m^3^/day
**Number of working hours per day**	8 h/day
**Number of working day per year**	302 day/year
**direct cost**	
**Raw material consumption**	9.32 $/m^3^
**Reagents**	3.57 $/m^3^
**Labors charge**	37.5 $/m^3^
**Energy consumption cost**	0.008 $/^m3^
**Mechanical instruments**	15 $/m^3^
**Electrical instruments**	9 $/m^3^
**indirect cost**	
**Maintenance cost**	12 $/m^3^
**Others cost**	7 $/m^3^

## Data Availability

The data presented in this study are available on request from the corresponding author.
